# A Review of the Construction of Nano-Hybrids for Electrochemical Biosensing of Glucose

**DOI:** 10.3390/bios9010046

**Published:** 2019-03-25

**Authors:** Razia Batool, Amina Rhouati, Mian Hasnain Nawaz, Akhtar Hayat, Jean Louis Marty

**Affiliations:** 1 Interdisciplinary Research Centre in Biomedical Materials (IRCBM), COMSATS University, Islamabad, Lahore Campus 54000, Pakistan; raziabatool@cuilahore.edu.pk (R.B.); mhnawaz@cuilahore.edu.pk (M.H.N.); 2 Ecole Nationale Supérieure de Biotechnologie, Constantine 25100, Algeria; amina.rhouati@gmail.com; 3 BAE: Biocapteurs-Analyses-Environnement, Universite de Perpignan ViaDomitia, 52 Avenue Paul Alduy, 66860 Perpignan CEDEX, France

**Keywords:** nano-hybrids, nano-transducer surface, glucose oxidase, electrochemical biosensor, glucose detection

## Abstract

Continuous progress in the domain of nano and material science has led to modulation of the properties of nanomaterials in a controlled and desired fashion. In this sense, nanomaterials, including carbon-based materials, metals and metal oxides, and composite/hybrid materials have attracted extensive interest with regard to the construction of electrochemical biosensors. The modification of a working electrode with a combination of two or three nanomaterials in the form of nano-composite/nano-hybrids has revealed good results with very good reproducibility, stability, and improved sensitivity. This review paper is focused on discussing the possible constructs of nano-hybrids and their subsequent use in the construction of electrochemical glucose biosensors.

## 1. Introduction 

Recently, efforts have been made towards the integration of nanomaterials in the construction of electrochemical transducer platforms. Consequently, nanomaterials have been employed to serve various functions in the design of biosensors [1]. Biosensors were first introduced by Leland Clark in 1962 [2]. A biosensor is a compact analytical device which relies on the immobilization of bio-molecules on a transducer surface. Numerous studies have been performed which have aimed to develop new biosensor assemblies and immobilization schemes based on the integration of innovative materials [3]. An ideal biosensor must be fast, cost-effective, and stable for sensitive and selective detection of target molecules. Among the different types of biosensors, electrochemical biosensors have gained special attention in the scientific community [4]. The ability of electrochemical biosensors to replace expensive and bulky analytical apparatuses has motivated scientists to investigate this arena by experimental and theoretical mechanisms. 

Electrochemical biosensors are classified into two groups: amperometric and potentiometric sensors. Amperometric biosensors are based on the redox output electrochemical signal under a defined potential. The amperometric method is the commonly used method in the design of electrochemical biosensors. Amperometric biosensors function by the production of a current when a potential is applied between two electrodes and the analyte undergoes, or is involved in, a redox reaction that can be followed by measuring the current in an electrochemical cell. Potentiometric biosensors make use of ion-selective electrodes in order to transduce the biological reaction into an electrical signal. In the simplest terms this consists of an immobilized enzyme membrane surrounding the probe from a pH-meter, where the catalyzed reaction generates or absorbs hydrogen ions. The analytical figures of merit of an electrochemical biosensor are associated with the properties of the transducer surface. For example, variation in the ionic condition and pH of detection media can alter the response of electrochemical biosensors, especially in the case of bio-affinity based biosensors [5]. The field has received considerable attention in recent years but there is a lot to address to improve the analytical performance of electrochemical biosensors. In this context, researchers have functionalized the transducer surface with nanomaterials to overcome the aforementioned problems [6]. Similarly, nanomaterials are known to amplify the electrochemical response in the design of biosensors. Nanomaterials are characterized by large surface area, improved catalytic properties, and ease of surface modification with a variety of functional groups. Their large surface area permits the efficient immobilization of the biomolecule on the transducer surface, resulting in enhanced sensitivity of the sensors. Furthermore, nanomaterials are known to promote the electron transfer rate of the transducer surface. Different nanomaterials have been widely explored to design sensors of interest for a large number of applications. These nanomaterials include but are not limited to metal/metal oxide, carbon-based, and composite nanoparticles. Different morphologies, sizes, shapes, and compositions of nanomaterials are expected to offer a major role in performing various functions in sensing systems. However, controlled synthesis of nanomaterials is crucial to achieving the desired functions. 

## 2. Role/Function of Nanomaterials in Electrochemical Biosensors

Nanostructures of many materials have been employed in the construction of electrochemical biosensors, such as oxide nanoparticles, metal nanoparticles, semiconductor nanoparticles, and composite nanoparticles or nano-hybrids. Although nanomaterials display different functions in electrochemical bio-sensing schemes according to their distinctive properties, the basic role of nanomaterials can be generally categorized as follows:

(1) Immobilization support for enzyme
(2) Nanomaterials as mediator
(3) Nanomaterials as signal amplifier

### 2.1. Immobilization Support for Enzyme

Enzyme immobilization is an important part of the construction of high-performance electrochemical biosensors. It affects bioactivity as well as the loading of enzymes. Numerous approaches have been considered to attain efficient enzyme immobilization including enzyme incorporation into various matrixes and the covalent binding of enzymes onto the surface of the substrate. The advanced construction of nanomaterials offers possibilities regarding the modification of properties, hence improving their application as well as function in enzyme immobilization. 

Different types of nanomaterials with variable shapes, sizes, and compositions have been developed and applied as unique electrode materials for required enzyme immobilization [7]. Direct loading of biomolecules onto bare surfaces of materials can lead to loss and denaturation of bioactivity behavior. However, the loading of biomolecules onto nanomaterial surfaces maintains their bioactivity due to nanomaterial biocompatibility. Meanwhile, most of nanomaterials have surface charges so that they can easily electrostatically adsorb biomolecules with various charges. Similarly, nanomaterials such as gold can immobilize proteins via covalent interactions among cysteine residues and amine groups of proteins and gold atoms [8,9]. 

**Figure 1:**
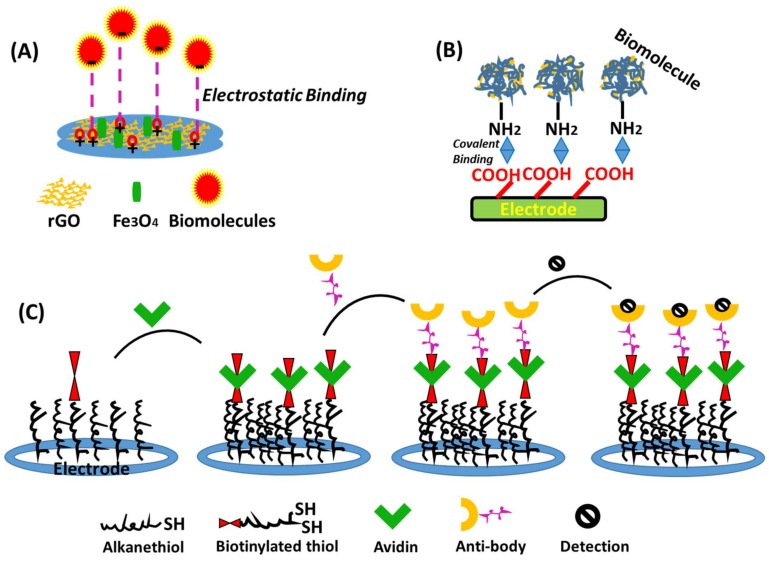
Overview of nanomaterial-based transducer surfaces in electrochemical biosensors; (**A**) Electrostatic binding; (**B**) covalent binding and (**C**) affinity binding.

The current growth in nanotechnology has opened up new prospects for the immobilization of enzymes through the synthesis of nano-composites or nano-hybrid materials. Figure 1 provides an overview of nanomaterial-based transducer surfaces employed in the construction of electrochemical biosensors.

### 2.2. Nanomaterials as Mediators

The electrical connection among biomolecules and the surface of the electrode is an important parameter in the assembly of electrochemical biosensors [10]. In an effort to increase the electron transfer rate between the electrode surface and biomolecules, metallic nanomaterials are being used which are capable of enhancing the electron transfer rate due to their metallic properties. Metallic nanomaterials play a significant role as a mediator in electron transfer. The generally used mediators are substituted by the metallic nanomaterials mediator. However, the role of such metallic nanomaterials mediators has been extensively explored in the case of amperometric biosensors. 

The electron flow rate among biomolecules and electrode surfaces can also be facilitated by the integration of some non-metallic nanomaterial mediators like oxide nanomaterials. The conductive behavior of nanomaterials as well as the arrangement among proteins and nanomaterials is directly associated with the enhancement of the electron transfer rate [11]. Gold nanoparticles have been used to improve the electron flow rate among biomolecules and the electrode surface in the assembly of an enzyme-based biosensor [12]. Moreover, silver nanomaterials also have better conducting properties which can be used to improve the electron flow rate among electrode surfaces and biomolecules. Silver nanoparticles in combination with pyrolytic graphite electrodes have been used as an electron transfer medium of biomolecules and electrode surfaces [13]. Nano-hybrids used as mediators have better conducting properties which enhance the electron flow between the electrode surface and biomolecules [14,15,16].

**Figure 2:**
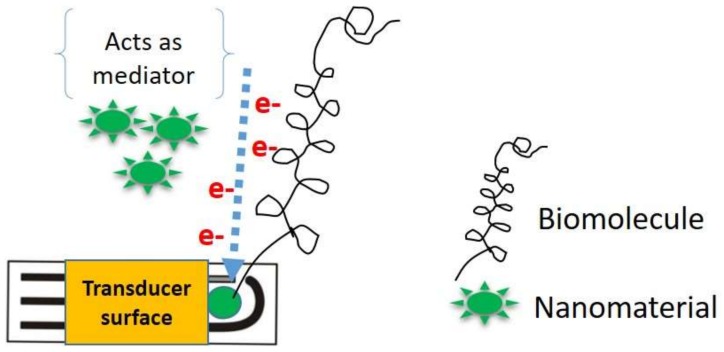
Nanomaterials as mediators in electrochemical biosensors.

The enhancement of the flow of electrons not only depends on nanomaterial conductivity but also on the arrangements among the biomolecules and nanomaterials. Well-defined and ordered arrangements of nanomaterials have been proven to be a fascinating approach to the designing of biosensors with improved electron flow properties. The well-organized arrangement of nanomaterials allows for the fast movement of analytes on the transducer surface, enhancing catalytic and sensing responses. The electrical properties of nanomaterials can be shaped in the desired way by reduction of the spatial dimension or the confinement of these structures in a precise crystallographic direction. The properties of nanomaterials are linked in particular, with the properties of nanomaterials mainly linked to different origins via a large fraction of surface atoms, large surface energy, spatial confinement, and reduced imperfections. These properties can be varied in accordance with the size, shape, or extent of agglomeration. Furthermore, the well-defined arrangement of nanomaterials facilitates uniform attachment of bioreceptors on the transducer surface, eliminating the random output current signal [10]. Figure 2 represents the function of nanomaterials as mediators in the construction of electrochemical biosensors.

### 2.3. Nanomaterials as Signal Amplifiers

**Figure 3:**
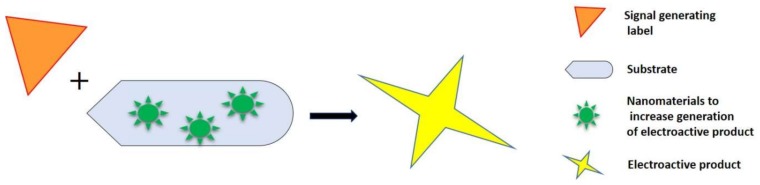
Integration of nanomaterials towards signal amplification of electrochemical biosensors.

Numerous studies have been focused on exploring novel methodologies to realize the ultrasensitive detection of analytes. These approaches comprise the integration of novel labels like redox complexes and metal ions, etc., and the integration of enzyme and mass spectroscopy supported signal amplification processes [17]. While these approaches have enhanced analytical performance, they are complex and have the drawbacks of high cost and derivatization. The leading methodology used to increase analysis sensitivity is the use of nanomaterial-based signal amplification formats. Nano-signal amplification has gained significance in achieving improved sensitivity for analyte detection. In the design of electrochemical biosensors, various nanomaterials including carbon nanomaterials, metal nanoparticles, semiconductors, and nanowire nanomaterials have been incorporated as electrochemical signal amplifiers [18]. Nanomaterials have been integrated to attain the attachment of enzymes on the transduction platform to enhance the signal of bio-recognition events as well to ensure an electrochemical reaction [19]. In signal amplification, nanomaterials are commonly used as catalysts to generate an electrochemical signal for the high loading of signal tags. The catalytic properties of nanomaterials can be well combined with the features of bio-receptors to fabricate highly sensitive electrochemical biosensors [20]. The hybridization of different nanomaterials has been integrated in the construction of electrochemical biosensors to achieve improved electronic properties and to retain the catalytic characteristics. Various morphologies and combinations of oxides and metals have been explored in this direction [21]. Figure 3 depicts the integration of nanomaterials towards signal amplification of electrochemical biosensors. 

## 3. Electrochemical Biosensors for Glucose Detection 

### 3.1. Glucose Monitoring

The detection of blood glucose levels for the continuous care of patients with diabetes is performed using numerous glucose biosensors. Since the occurrence of diabetes is increasing, new glucose biosensors based on point of care testing (POCT), such as wearable sweet-based sensors and non-invasive methods for glucose monitoring, have been reported during the last few years [22,23,24]. One such example is the integration of stainless steel pins based on transducer electrodes to fabricate easy to use and portable amperometric glucose biosensors [25]. The importance of glucose sensing based on POCT has been extensively established by medical professionals and by patients. Furthermore, optical sensors have been developed for the visual detection of glucose. Optical detection can be good for qualitative analysis but is characterized by poor sensitivity for quantitative measurements. Similarly, conventional analytical methods require complex instruments and skilled personnel to operate them and they are unsuitable for on-site analysis. 

Glucose biosensors have been developed for consistent, fast, accurate, and easy to operate monitoring of glucose levels. Additionally, non-enzymatic nanomaterial-based optical and electrochemical glucose diagnosis has been discussed in the literature. Our group has recently reviewed the use of biomimetic nanomaterials in the detection of glucose [26]. Nanomaterials have been employed to replace the enzyme in the monitoring of glucose [27]. Non-enzymatic electrochemical sensors employing nano-hybrids such as gold/copper oxide [28], MoS2-Au@Pt [29], nickel in conjugation with multi-walled carbon nanotubes [30], and palladium nanoparticle-graphene [31] have been reported in the literature. Although such constructs are non-enzymatic, they are characterized by low selectivity, transducer surface fouling and the non-specific adsorption of the sample matrix. Despite remarkable developments in glucose monitoring, there are still some limitations associated with the fabrication of consistent glucose analysis. The ADA claims the precision of a blood glucose POCT assay to be <5% of the obtained value. However, many POCT assays do not fulfil this standard. Biosensor approaches are less defined and less precise than assays employed in laboratory analysis [32]. With this in mind, the past nearly 50 years have observed incredible development in the growth of electrochemical glucose devices. Well-directed research on novel sensing ideas, combined with several nanotechnological inventions, has therefore unlocked the possibility of extensive use of electrochemical methods. With advancements in nanotechnology, various nanomaterial-based single/multi components have been explored with unique characteristics including low cost, large surface area, robustness to harsh conditions, and ease of manipulation to construct electrochemical glucose biosensors. In this context, researchers have reviewed the integration of different nanomaterials in the construction of glucose biosensors. For example, Zhu et al. published a review paper on carbon nanomaterial integration in electrochemical biosensors for glucose detection. This work aimed to discuss synthesis procedures, recent sensing approaches, and nonenzymatic hybrid electrochemical sensors [33]. Similarly, Rahman et al. reviewed the role of various nanomaterials such as ZnO, Cu(I)/(II) oxides, MnO_2_, TiO_2_, CeO_2_, SiO_2_, ZrO_2_, and other metal-oxides in glucose biosensors. Authors have summarized the working principles of these different types of nanomaterial-based electrochemical glucose biosensors [34]. However, the objective of the present work is to discuss the possible constructs of nano-hybrids and their subsequent use in the fabrication of electrochemical glucose biosensors. 

The next section of this review paper categorizes different types of nanomaterials integrated in the construction of electrochemical glucose biosensors. 

### 3.2. Carbon-Based Nano-Hybrids and Nano-Composites

**Figure 4:**
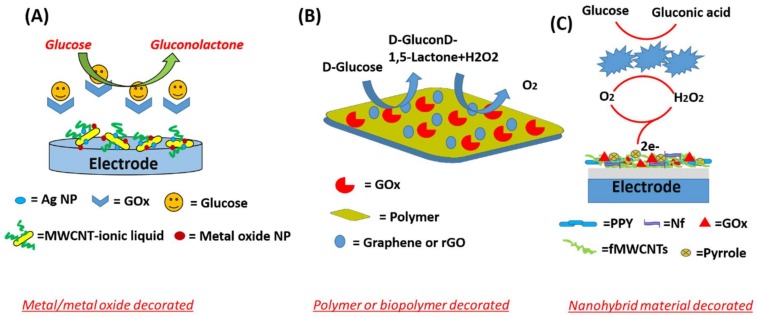
Illustration of types of carbon-based nano-hybrid and nano-composite materials in electrochemical glucose biosensors (**A**) AgNP/MWCNT; (**B**) rGO/Polymer and (**C**) fMWCNT/Polypyrrole.

Carbon materials, due to their intrinsic electrochemical features of relative chemical inertness, a wider potential window in aqueous media, and lower background current, have received much attention in recent years. Based on their superior electrochemical properties, various electroanalytical applications of carbon materials have been explored [35,36]. Figure 4 illustrates different types of carbon based-nano-hybrid and nano-composite materials for employment in the construction of electrochemical glucose biosensors.

Similarly, in the quest for highly selective and sensitive monitoring, carbon-based materials have gained primary attention in glucose analysis. These materials have diversity in their morphology, consisting of 1D (carbon nanotubes), 2D (graphene and graphitic carbons), and zero dimensional fullerenes and carbon quantum dots, and hence are characterized with multifarious electrochemical properties [36,37]. Tiwari et al. presented an interesting statistical analysis illustrating the leading role of carbon materials (mainly after the discoveries of carbon nanotubes and graphene) in the domain of sensors and biosensors [38]. For instance, carbon nanotubes, due to their higher surface to volume ratio and ability to mediate fast electron transfer kinetics, are the most attractive materials in the architecture of electrochemical transducers [39]. In addition, Wang et al. used single-layered reduced graphene oxide to functionalize glucose oxidase for glucose detection by realizing the direct electron transfer between the electrode and the enzyme [40]. In glucose oxidase based detections, graphene overcomes proteinic hindrance covering the redox active centers of the enzyme and facilitates direct electron flow [41].

To further tune up the electrochemical properties of carbon materials and to get the best of their intrinsic features, tailoring and development of chemical and physical changes to the surfaces of these materials has recently attracted great attention. For example, chemical doping of heteroatoms (N, S, B, P, and Se, etc.) within graphene and carbon nanotubes could enhance the electrocatalytic properties of these carbon materials. Mansour et al. recently investigated the glucose detection performance of microwave treated N-functionalized doped graphene [42]. Chen et al. prepared nitrogen- and sulfur-based dual-doped graphene via a two-step solvothermal method for improving the electrocatalytic sensing performance towards glucose [43]. Fan et al. utilized carbon-foam-supported N-doped CNT to develop an efficient and high-performance glucose biosensor [44].

To further improve the device performance and reduce the signal-to-noise ratios of carbon-based biosensors, carbon materials are integrated with various types of polymers, ionic liquids, biomolecules, and metals and metal oxides either in the form of nano-hybrids or nano-composites. In this vein, Zhang et al. reported a direct-electron-transfer-assisted electrochemical biosensor based on an N-doped carbon nanosphere@nanofibers composite for glucose detection [45]. Chen and co-workers developed a simple glucose sensor based on a one-step electrodeposited chitosan-CNT [46]. The presence of chitosan, a biocompatible polymer, increased the scope of application of such a composite without compromising its electrocatalytic properties. In addition, Yang et al. prepared modified electrodes with sonication-induced chemically modified graphene and an ionic liquid hybrid structure for sensitive glucose detection [47].

Keeping in mind the dependence of electrochemical performances of biosensors on pore size and surface area of the composite structure, Yang et al. prepared nano-hybrids of polyoxometalate (POM) deposited with functionalized graphene oxide on polymer-blended ionic liquid (PIL) [48]. Highly efficient electrocatalytic POM-based graphene hybrids exhibited fast electron transfer and high electrocatalytic activity towards glucose with superior analytical parameters. Similarly, to enhance the film-forming properties of composite materials without compromising the electron transfer capability, several groups have used conducting polymers to synthesize carbon material based nano-composites and have demonstrated their glucose detection abilities. For example, graphene-coated paper was immobilized with poly(9,9-di-(2-ethylhexyl)-fluorenyl-2,7-diyl)-end which was further capped with 2,5-diphenyl-1,2,4-oxadiazole (PFLO) to provide channels for enzyme anchoring via physical adsorption [49]. Hui et al. electrodeposited GO-doped poly(3,4-ethylenedioxythiophene) (PEDOT), a well-known conducting polymer, on an electrode surface, followed by in-situ growth of Ni nanoparticles, to exhibit outstanding electrooxidation of glucose [50]. Sheng et al. prepared bimetallic nanoparticles decorated on an in-situ polymerized polypyrrole and reduced graphene oxide composite as a supporting substrate for glucose detection. The as-prepared nano-composite displayed excellent electrocatalytic performance for glucose oxidation [51]. Similarly, Xu et al. demonstrated that a graphene/polyaniline/gold nanoparticle-based non-enzymatic biosensor outperformed the starting materials in the electrochemical diagnoses of glucose with the intrinsic benefit of biocompatibility and stability [52]. Zhang et al. demonstrated that in situ reduction of a Cu precursor on polyaniline nanofibers can be used to adsorb different contents of graphene. The direct interaction of N atoms of PANI with Cu NPPs and the presence of a graphene structure corroborates the enhanced electron transfer properties into the nano-composite resulting in highly sensitive detection of glucose [53]. The employed materials resulted in efficient immobilization of the glucose oxidase which subsequently permitted the sensitive detection of glucose. 

Another class of carbon-material-based nano-hybrids and composites is the formation of composite structures with a variety of metals and metal oxides with a particular interest in the detection of glucose. The additional factor of electrocatalytic behavior of metals/metal oxides for the oxidation of glucose is the key feature of such devices. Yan et al. simultaneously reduced graphene oxide and synthesized CuS nanoflakes via hydrothermal treatment to give copper sulfide nanoflakes in combination with reduced graphene oxide (rGO/CuSNFs). Subsequently, this nano-composite demonstrated excellent selectivity and sensitivity towards non-enzymatic detection of glucose [54]. Likewise, in lieu of a solid support, carbon-based material can also be used as a flexible substrate for further modifications of glucose detection. For instance, recently, Jianan et al. demonstrated that Co_3_O_4_-decorated carbon cloth is an efficient glucose detection material [55]. These different Co_3_O_4_ crystal facets, grown directly on the carbon interface via simple hydrothermal treatment of carbon cloth, demonstrated a superior limit of detection (0.012 μM) with a wider linear range (0.5–1000 μM) for non-enzymatic glucose detection. In general, several nano-hybrids composed of carbon nanotubes and graphene derivatives have been reported for glucose detection. In Table 1, a few recent examples are listed with respect to their limit of detection and linear range parameters.

### 3.3. Metal/Metal Oxide-Based Nano-Hybrids and Nano-Composites

Metallic nanoparticles (MNPs) have been widely incorporated as immobilizers in biosensor fabrication. They have shown high surface area, good compatibility, high chemical stability, and good conductivity [67]. Different MNPs (e.g., Cu, Ir, Zn, Ag, Fe, Pd, Au, Pt, and their different alloys) have been used for the immobilization of glucose oxidase (GOx) to construct electrochemical-based glucose biosensors. They provide an enhanced surface area and easier but faster electron transfer from biological components to electrodes, eventually improving the detection signal [68]. Moreover, by associating two or more metals in a nano-hybrid, several favorable properties will be combined, leading to technique characteristics which cannot be attained by one component. For example, the low catalytic activity of gold could be compensated by Pt alloys to enhance the catalytic behavior synergistically [69]. Furthermore, incorporation of films and/or polymers (chitosan, nafion, or mesoporous silica, etc.) into a metallic nano-composite increases the surface stability, prevents enzyme leakage, and protects its native conformation [70]. Table 2 summarizes glucose biosensors fabricated by metallic nano-hybrids that have been so far reported in the literature. 

Owing to their unique characteristics, gold nanomaterials are the most widely used MNPs in glucose electrochemical biosensing. Numerous nano-hybrids of gold have been reported, in which gold-modified electrodes exhibit an excellent affinity for GOx, thus increasing the immobilization yield. Wei et al. synthesized a nano-composite of Au nanocrystals grown on the surface of Zinc oxide nanorods. ZnO nanostructures have been chosen for their high surface-to-bulk ratio, higher biocompatibility, electrochemical activity, and stability at normal physiological conditions. Indeed, the high isoelectric point (IEP) of ZnO provides a positively charged environment for low IEP enzyme immobilization of, e.g., glucose oxidase [71,72]. The constructed nano-composite has been used to entrap GOx and provides an enhanced electron transfer from biomolecules to electrode. The resulting biosensor has exhibited excellent analytical performance within a short time [73]. ZnO nanorods have been also combined with AuNPs for electrostatic adsorption of GOx. The analytical performances of thusly formed glucose biosensors were electrochemically investigated by cyclic voltammetric responses and amperometric signatures. The results showed a better analytical performance compared to ZnO nanorod-based biosensors. This has been attributed to the greater surface area and excellent electrocatalytic performance of the AuNP-modified ZnO nanorods [74]. Moreover, Fang et al. investigated the direct electrochemistry of GOx immobilized on functionalized AuNPs with hierarchically 3D ZnO nanostructures. The synthesized nano-composite favored enzyme immobilization and easy access to glucose molecules, which facilitated the efficient catalysis of glucose oxidation and fluent electron transfer for GOx. The reported nano-hybrid combines the greater surface area of nanoarchitecture and the superior conductivity of AuNPs. Hence, it generates a synergistic effect and enhances the performance of the developed biosensor. However, the exhibited LOD was much higher than that of the two biosensors described above [75]. Mandal et al. described the creation of gold nanoelectrode ensembles to enhance the sensitivity of electrochemical glucose biosensors. The authors employed ZnO nanorods to increase the active reaction area from a two dimensional to a three dimensional area, thus enhancing the electron transfer. The results indicate that the gold nanoelectrode ensemble-based working electrode demonstrates a higher current response and faster response time compared to previously reported enzymatic electrochemical sensors [76].

**Figure 5:**
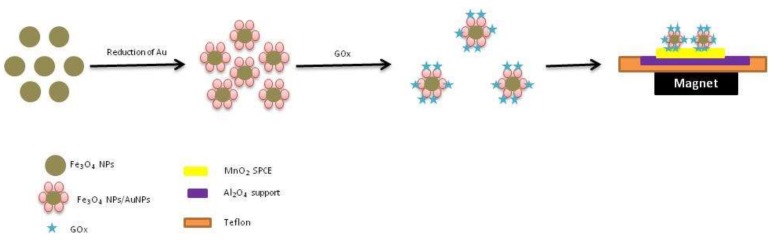
Schematic representation of flow-injection-based amperometric glucose biosensor. Glucose oxidase was immobilized on Fe_3_O_4_ core nanoparticles in association with Au seeds [78].

The assembly of gold with silver has been also reported for GOx immobilization; it is known that Ag has very good catalytic properties while Au has good conductivity. The Ag-Au nano-assembly has been prepared in reverse micelles where enzymes exhibit higher activity than in aqueous systems. Electrochemical studies have shown the role of Au-Ag nanoparticles in enhancing the electron transfer, stability, and sensitivity of glucose biosensing [77]. In a flow injection mode, amperometric glucose detection based on the immobilization of GOx onto Au seeds has been reported. The nano-composite was immobilized on a screen-printed interface with manganese oxide (MnO_2_) (Figure 5) via drop casting. An enhanced sensitivity has been noted owing to the synergistic effect of manganese oxide as a mediator in combination with Fe_3_O_4_NPs [78].

Immobilization of GOx on chitosan-metallic NP bio-composites has been widely described. Chitosan provides a biocompatible environment for GOx along with the power to withstand pH- and temperature-related harsh physiological conditions. In parallel, AuNPs enhance the stability, catalytic activity and affinity for GOx, and facilitate the electron transfer for hydrogen peroxide detection [79]. The simple electrochemical deposition of chitosan-GOx-AuNPs biofilms, one step electrodeposition, and layer by layer assembly protocols have been reported for glucose detection purposes. The three methods have showed excellent analytical performance, high stability, and fast electrochemical response times. However, the best sensitivity has been obtained in the one step electrodeposition method [79,80,81]. In another report the same composite was deposited on a Prussian-blue-modified Pt reference electrode. Improved analytical performances have been obtained owing to the synergistic catalysis characteristics of Prussian blue [82].

Likewise, iron oxide NPs have also been used in combination with chitosan to cast nano-composite-based films on indium-tin-oxide-based electrodes. The support was used to adsorb GOx for the electrochemical determination of glucose. In the reported nano-hybrid, chitosan prevents the aggregation of Fe_3_O_4_ NPs without affecting their electrocatalytic properties [83]. In another report, Benvenuto et al. immobilized GOx/chitosan onto nanotube arrays of TiO_2_ which had already been modified by Au and Prussian blue. They grew TiO_2_ nanotube arrays directly via anodic oxidation, followed by a coating of thin Au film. The authors demonstrated that their larger surface area and good uniformity make TiO_2_ nanotubes an excellent immobilization matrix, while the Au thin film greatly incorporated increased electrical activity into the TiO_2_ nanotube arrays [84].

Conducting polymers, including polypyrrole (PPy) and polyaniline (PANI), have also been combined with metallic NPs as GOx immobilizers in glucose diagnostics. They are used as a matrix which introduces common properties for biomolecule immobilization [85]. Electroactive nano-composites of AuNP-polyaniline nanofibers have been synthesized to immobilize GOx for the amperometric detection of glucose. This encapsulation strategy eventually hinders branching of polymeric structures and promotes lateral growth of polymers (PANI long chains) which eventually enhance the entrapped amount of GOx [86,87]. High-density platinum NPs have also been homogeneously loaded onto a nanostructured matrix of the PANI hydrogel. The resulting nano-composite exhibited a high conductivity; the porous PANI improves the enzymatic immobilization rate while PtNPs catalyze the generated H_2_O_2_ decomposition [88]. In another report, German et al. developed a biosensor for glucose detection based on a film of gold nanoparticles covered by polypyrrole. Due to their excellent electrical properties and π-π conjugation, conducting polymers can act as mediators and ultimately facilitate the electron transfer to the electrode [89]. A platinum nanocluster embedded polypyrrole nanowire (PPy-Pt)-based composite has also been synthesized by Lin’s group. They also immobilized GOx on an electropolymerized non-conducting poly(*o*-aminophenol) (POAP) film which had already been deposited onto the PPy-Pt based electrodes. The authors demonstrated that the nano-composite provided a suitable environment for GOx and enhanced the sensor stability [90]. Later, poly(vinyl alcohol)/poly(ethyleneimine) nanofibers (PVA/PEI NFs) were decorated with AuNPs and used as enzymatic immobilizers. This strategy was proposed to increase the conductivity of electro-spun polymer/enzyme NF mats in a simple but effective manner. In this report, a density of particles was observed and attributed to hydrogen bonding and characteristic ionic interactions due to NH_2_ of PEI and COOH of citrate-stabilized Au NPs. Electrochemical impedance measurements have indicated the applicability of biosensor for glucose detection with higher sensitivity and a larger dynamic range, in addition to storage stabilities [91]. Azak et al. reported dithizonepyrrole (DTP)-type monomers and used them for efficient detection of glucose. Pi-conjugated materials are characterized by good charge transport properties, homogeneous film forming behavior, and tunable physical and optical characteristics [92].

Due to their stability, biocompatibility, porous structure and enlarging surface, dendrimers have been also combined with metallic nanomaterials for biosensor fabrication. For example, polyamidoamine (PAMAM) contains excessive amine functionalities which facilitate enzyme immobilization. Several reports have described the use of AuNPs-PAMAM composites as GOx immobilizers and their application in glucose detection [93,94]. 

Room temperature ionic liquid possesses many merits including high conductivity, higher thermal stability, and lower electrochemical potential. Li et al. reported the direct electrochemistry of GOx immobilized on a gold nano-particle/*N*,*N*-dimethylformamide (DMF)/ionic liquid composite film on a glassy carbon electrode. Electrochemical studies showed that the constructed nano-hybrid was capable of GOx immobilization to give a pair of stable redox peaks with reversible character. Without any of these materials, the redox peaks of GOx become smaller and more unstable [95].

Recently, nano-hybrids based on one dimensional materials and nanoparticles of noble metals, with a synergistic effect, have found many applications in biosensing. In comparison with spherical nanoparticles, nanowires possess a number of unique electronic and physical properties due to their anisotropic characteristics during electrochemical reactions at electrode surfaces. Indeed, they provide a high loading efficiency and a compatible microenvironment for enzyme immobilization. Wang et al. demonstrated that the synergistic effect of Pb nanowires and AuNPs improved the electrochemical biosensing of glucose through excellent electrocatalytic activity towards glucose [96]. Advantages of 2D nanomaterials have been also explored; Su et al. constructed a glucose biosensor based on GOx immobilization on molybdenum disulfide (MoS_2_) nanosheet modified GCE decorated with AuNPs. The nano-composite showed excellent electrocatalytic activity, which was incorporated by molybdenum disulfide and Au characteristic behaviors [97]. 

In addition to the Zn-AuNPs nano-hybrids discussed above, zinc materials have been used to construct other nano-composites for glucose enzymatic sensing. Zhao et al. combined the high surface area and nanoporous structure of ZnO-based nanoclusters with the electrocatalytic activity of cobalt for reducing oxygen. The results showed that thin films of ZnO:Co nanoclusters bearing nanoporous structures and crystallites had potential applications as superior enzyme immobilization platforms [98]. Later, the synthesis and characterization of an NiO-doped ZnO nanorod composite and its subsequent applications in enzyme immobilization were investigated. Compared to ZnO nanorods, NiO-doped ZnO nanorods have been proven to have improved catalysis character of GOx toward glucose oxidation reaction [99].

### 3.4. Other Nano-Composites

**Figure 6:**
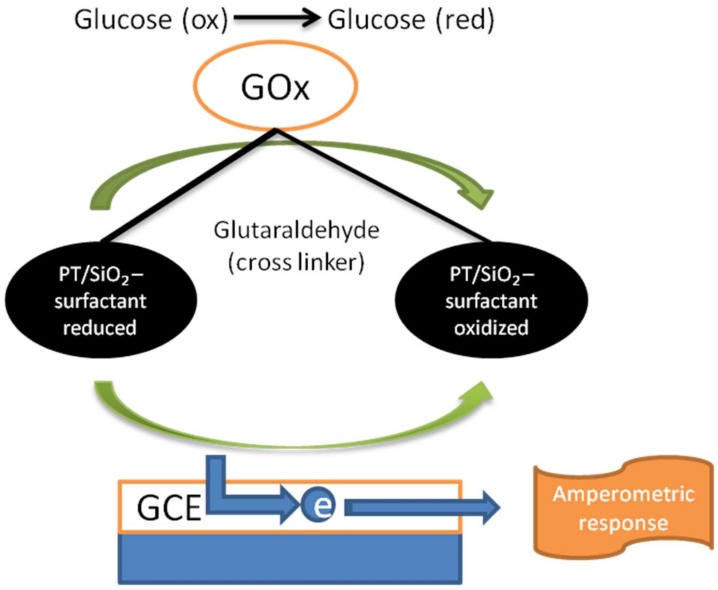
Representation of an amperometric glucose biosensor based on a polythiophene/SiO_2_ nano-composite in the presence of surfactants [101].

In the literature, the majority of glucose biosensors follow the protocol of immobilization of glucose oxidase on metallic/carbon nano-hybrids. However, some reports have described other nano-composites without metallic or carbon nanomaterials, such as SiO_2_. For instance, Uygun et al. prepared a conducting polythiophene/SiO_2_ (PT/SiO_2_) nano-composite in the presence of anionic/cationic/non-ionic surfactants to create a GOx-immobilized polymeric amperometric biosensor. The authors noted that the employed surfactants not only act as a useful surfactant but they also serve as a co-doping agent for PT. It increased the charge carriers in the composite matrix and the impacts on the electric conductivity and diagnostic properties of these composite materials (Figure 6). Using the developed biosensor, glucose determination was performed across a wide linear range, from 60 to 1.585 × 10^3^ µM [101]. In a recent study, Kadian et al. synthesized an inorganic/organic TiO_2_ NP and poly (3-hexylthiophene) (PHT) to allow GOx immobilization on an indium-tin-oxide (ITO) based electrode. PHT has been used for its well-known conductivity (a field effect mobility of up to 0.1 cm^2^/Vs). The resulting sensor showed high specificity and selectivity for glucose detection in saliva samples with a fast analyte response time (<10 s) [102]. Another conducting polymer-based nano-composite has been reported by Yao’s group. A poly(methylene blue)-doped silica nano-composite was synthesized on a GCE based on a two-step process of electropolymerization. Moreover, the GOx molecules immobilized on the polymer nano-composites underwent a direct reversible electrochemical reaction. The authors demonstrated that the synthesized nano-composite facilitated the electron transfer from the biomolecules towards the electrode interface. The increased electrochemical signals were attributed to the higher biocompatibility of the SiO_2_ matrix and higher enzyme-loading properties due to the greater active sites of these nano-composites exposed for enzyme immobilization. Differential pulse voltammograms have shown a detection limit of 3 µM and linearity over a glucose concentration range of 10 to 1.11 × 10^3^ µM [103]. In another report Zhao et al. combined silica with phytic acid, which is a naturally derived material known for its biocompatibility and conductivity [104]. In this work, the authors constructed a silica–phytic acid nano-composite via reverse microemulsion and electrostatic binding protocols. Then, glucose oxidase was immobilized onto the nanoconstruct based on a GCE. Response studies towards glucose were carried out via DPV, which demonstrated a detection limit of 12 µM [105].

Finally, a biosensor was fabricated from drop casting of chitosan–polypyrrole nano-composites onto a carbon electrode as superior transducer materials for glucose detection. The nano-composites combined the electroconductive properties of polypyrrole, the film-forming properties of biocompatible CS, and the surface area-related advantages of nanoparticles. Thus, it exhibited an enhanced electron transfer capability from glucose oxidase to the electrode interface. The fabricated biosensor exhibited a faster amperometric response time (5 s), a lower LOD (15.5 µM) and a wider linear range (500–0.147 × 10^6^ µM). Indeed, the higher biocompatibility and film-forming properties of nano-composites improved the reproducibility and stability of the biosensor [106]. Table 3 provides an insight on electrochemical glucose biosensors based on nanocomposites other than metal oxides and carbon nanomaterials.

## 4. Conclusions and Prospective Research 

Nano-electrochemical biosensors have moved scientific research into a novel direction with improved analytical features of merit. Different types of nanomaterials have been integrated into the design of electrochemical biosensors for glucose detection. This review paper has summarized the possible role of nanomaterials in the construction of electrochemical biosensors, with a particular interest in nano-electrochemical glucose biosensors. Nanomaterial-based electrochemical biosensors provide better analytical performance in term of fast response time, cost effective transducer surface construction, and stability over an extended period of time. Much effort has been focused in this direction but still there are lot of parameters that need to be addressed in the future. Research has been focused on combining the properties of multi nanomaterials within a single system. This has led scientists to design nano-composites/nano-hybrids which are subsequently employed in the construction of electrochemical biosensors. However, it is highly desirable to retain the properties of the basic materials during the synthesis of multicomponent systems. Contributing nanomaterials may partially lose their specific characteristics in combination with other materials. Future research may focus on the exploration of novel materials, the combination of novel materials to design multicomponent systems, and controlled and reproducible novel synthesis procedures. In the same vein, immobilization of nanomaterials on transducer surfaces to deposit uniform layers is of vital importance to obtain repeatable and reproducible results. Drop casting is mainly used to form nanolayers on the transducer surface, which is highly prone to random surface thicknesses and ultimately affects the performance of electrochemical biosensors. Therefore, alternative immobilization methodologies for nanolayer formation need to be investigated in the construction of electrochemical biosensors. 

## Figures and Tables

**Table 1:** Carbon material-based nano-hybrids and nano-composites for the electrochemical detection of glucose.

Sr#	Nanomaterials/Composites	Details	LOD (μM)	Linear Range (μM)	Ref.
1	PVC–ZnO–MWCNT	Simple mixing/drop casting/GCE	-	20–17.8 × 10^3^	[56]
2	Bioengineered Nf-GOx-fMWCNTs-PPy/Pt electrode	In situ electrochemical polymerization/Pt electrode	5.0	5.0–4.1 × 10^3^	[57]
3	Graphene-gold	Thermal annealing-freeze drying/GCE/Graphene aerogel	4.0	0.01–16 × 10^3^	[58]
4	rGO-SWCNT-Au	One pot reduction/GCE/in situ growth of Au NPs	0.0022	0.00001–80 × 10^3^	[59]
5	AuNP/GONR/CS	Graphene oxide nanoribbons as a supporting matrix/carbon sheet/drop casting	-	0.5–10 × 10^3^	[60]
6	Ni(OH)_2_-CNT-PVDF	Simple dispersing/GCE	23	0.25 × 10^3^–39.26 × 10^3^	[61]
7	Ni_3_S_2_/carbon nanotube	Glucose-assisted hydrothermal method/Ni foam electrode	3.3	30 × 10^3^–500 × 10^3^	[62]
8	CNT/Si-SiO_2_	Magnetron sputtering/simple dispersion/silica substrate electrode/CNT/Ni working electrode	2.0	5–7 × 10^3^	[63]
9	ALD/CVD-assisted CNT–Ni	Carbon tetrabromide precursor and Au-assisted ALD/CVD procedure/glassy carbon electrode	2.0	5–2 × 10^3^	[64]
10	Ni–MWNT	Electrodeposition/glassy carbon electrode/drop casting	0.89	3.2–17.5 × 10^3^	[30]
11	Carbon-coated ZnO (ZnO@C)	Surface coating via hydrothermal process and CVD method/GCE	1000	1 × 10^3^–13.8 × 10^3^	[65]
12	Fe_3_O_4_/CG	One-step ball milling/EDC-assisted modification/Pt sputter-coated ITO glass	16.0	16–26 × 10^3^	[66]

**Table 2:** Electrochemical glucose sensors based on metallic nano-hybrids.

Sr#	Nanomaterials/Composites	Details (Substrate, Precursor, Electrode, and Synthesis Method)	LOD (µM)	Linear Range (µM)	Ref.
1	ZnO nanorods/Au nanocrystals	Facile hydrothermal route/enzyme entrapping/GCE	0.01	0.1–33	[73]
2	ZnO nanorods/AuNPs	hydrothermal and photo reduction method/fluorine-doped tin oxide	3	3–3 × 10^3^	[74]
3	AuNPs-3D ZnO	In situ reduction for Au NPs/drop casting/GCE	20	10^3^–20 × 10^3^	[75]
4	ZnO nanorods/Au	Hydrothermal growth of nanorods/Au-ITO electrodes	65	10–6.5 × 10^3^	[76]
5	Au seeds-Fe_3_O_4_ NPs	Enzyme chemisorption/SPCE/carbon substrate	100	200–9 × 10^3^	[78]
6	Chitosan-GOx-AuNPs	Electrochemical deposition/Au disk electrode	2.7	5–2.4 × 10^3^	[79]
7	Chitosan-AuNPs-GOx	LBL assembly/enzyme adsorption/Pt electrode/	7	500–16 × 10^3^	[80]
8	Chitosan-AuNPs film	Direct and facile electrochemical deposition method/GCE	13	0.5–1.3 × 10^3^	[81]
9	Chitosan-AuNPs	In situ incorporating glucose oxidase/electrodeposition of chitosan/GCE	0.69	1–1.6 × 10^3^	[82]
10	Chitosan-Fe_3_O_4_ NPs	Co-precipitation method/ITO/physically adsorbed enzyme	-	550–22 × 10^3^	[83]
11	Chitosan-TiO_2_-Au	Argon plasma coating/Ti substrate/electrodeposition	5	15–4 × 10^3^	[84]
12	PANI-AuNPs	Simple mixing/drop casting/GCE	0.5	1–800	[86]
13	PANI-AuNPs	Enzyme entrapment/simple mixing/drop casting/carbon rod electrode	-	100–150 × 10^3^	[87]
14	PANI-PtNPs	Hydrogel heterostructure/GCE	0.7	10–8 × 10^3^	[88]
15	AuNPs-polypyrrole	Adsorbed electron transfer mediator/solution casting/graphite rod electrode	24	100–50 × 10^3^	[89]
16	Pt-polypyrrole	Electrosynthesis, GCE/film deposition	0.45	1.5–13 × 10^3^	[90]
17	AuNPs-PVA/PEI NFs	Bioactive surface nanostructuration method/electrospun nanofibers of PVA-PEI/Au electrode	0.9	10–200	[91]
18	AuNPs-DTP	Dithionepyrrole-based conducting polymers/Au electrode	0.0986	50–1000	[92]
19	AuNPs-PAMAM	Electrocatalytic membranes/layer-by-layer/ITO electrodes/enzyme immobilization via cross-linkers	17	Up to 30	[93]
20	AuNPs-PAMAM	Self-assembled monolayer/dendrimers/Au electrodes	600	10^3^ –5 × 10^3^	[94]
21	AuNPs-ionic liquid	Enzyme entrapment in nanogold particles/composite film forming/GCE/	3.49	2–20	[95]
22	AuNPs-Pb nanowires	Pb decoration nanowires/matrix of bovine serum albumin/Pt electrode	2	5–2200	[96]
23	AuNPs-MoS_2_	MoS_2_ nanosheets assisted enzyme immobilization/without electron mediator/GCE	2.8	10–300	[97]
24	ZnO:Co nanoclusters	Nanocluster-beam deposition/cross-linking/PET plate electrode	20	-	[98]
25	NiO-doped ZnO nanorods	NiO-doped ZnO nanorods/enzyme immobilization/Pt electrode	2.5	500–8 × 10^3^	[99]
26	NiCo_2_O_4_@polyaniline core shell	Hydrothermal treatment/conducting polymer coating/GCE	0.3833	Up to 4.7 × 10^3^	[100]

**Table 3:** Electrochemical glucose biosensors based on nanocomposites other than metal oxides and carbon nanomaterials.

Sr#	Nanocomposites	Details	LOD (μM)	Linear Range (μM)	Ref.
1	PT/SiO2	Chemical oxidative polymerization/enzyme-immobilized polymers/enzyme immobilization via crosslinking/GCE	-	6–1585	[101]
2	ITO/TiO2/PHT/GOx	Solution deposition/ITO electrode	0.62	1–310	[102]
3	PMB@SiO_2_(nano)	Electropolymerization/glassy carbon electrode	3	0.01–1.11	[103]
4	PA-SWNTs/Pt	PA-SWNTs films/charged linker/Pt electrode	8	20–10,000	[104]
5	SiO_2_-PA	Reverse microemulsion and electrostatic binding/glassy carbon electrode	0.012	-	[105]
6	CS–PPy	Silicon–oxygen interaction/drop casting/glassy carbon electrode	0.15	5–147	[106]
